# Echinacoside Protects Against Dysfunction of Spermatogenesis Through the MAPK Signaling Pathway

**DOI:** 10.1007/s43032-021-00707-y

**Published:** 2021-08-23

**Authors:** Guifang Zhao, Ying Wang, Zengyan Lai, Lianwen Zheng, Donghai Zhao

**Affiliations:** 1 Department of Pathology, Jilin Medical University, No. 126 Jilin Street, Jilin, 130013 China; 2 Department of Ophthalmology, the Second Hospital of Jilin University, Changchun, China

**Keywords:** Echinacoside, Testicular tissue, Leydig cells of testis, mitogen-activated protein kinase

## Abstract

Dysfunction at various levels of spermatogenesis (SD) is one of the important causes of infertility in men of reproductive age and requires advanced treatment strategies. Increasing evidence suggests that the therapeutic effects of echinacoside (ECH) mainly depend on their capacity to inhibit cell death. This study aimed to explore the therapeutic potential of ECH in SD rat models. Treatment with ECH reverted the morphological changes observed in testes with spermatogenesis dysfunction. It improved total sperm number, decreased the sperm deformity rate, and increased the sperm forward motility rate. The level of glutathione (GSH) was significantly higher in ECH-treated mice, whereas the lactate dehydrogenase (LDH) and SOD activities were improved compared with those in the spermatogenesis dysfunction model. Moreover, the increased expression of p38 and JNK was partially reversed by ECH. The number of normal TM3 cells increased gradually in an *Echinacea* dosage-dependent manner, suggesting that ECH promoted the proliferation of TM3 cells. In addition, treatment with ECH partially reversed the increased expression of p38 and JNK in TM3 cells. ECH protects against oxidative stress damage by activating antioxidant enzymes and MAPK signaling-related factors (p38 and JNK). It suggested that treatment with ECH alleviated spermatogenetic dysfunction of testes in male mice and it could be a promising strategy for patients suffering severe SD.

## Introduction

According to the World Health Organization (WHO), approximately 15% of couples of childbearing age have infertility problems, the worldwide incidence of which is increasing annually [1–3].

Some studies have shown that lead pollution could induce spermatogenic cell apoptosis, affect sperm development, and decrease sperm concentration, resulting in spermatogenic disorders [4]. An increasing number of men are exposed to lead for short or long periods of time daily owing to their occupations [5]. To this day, Western medicine (e.g., gonadotropin replacement therapy, dopamine receptor agonists, and antioxidant supplements) has been used to treat spermatogenesis disorders [6], but in recent years, traditional Chinese medicines have been gradually developed [7, 8]. However, there have been few reports that clarify the mechanisms of action of Chinese medicine used to treat the induced spermatogenesis disorders.

Echinacoside (ECH), a natural phenylethanoid found in many medicinal plants, is the principal constituent of the phenylethanoid glycosides isolated from the traditional Chinese herb *Cistanche salsa* [9]. Recent studies have shown that ECH exhibited protective effects in a mouse model of lead acetate-induced spermatogenic dysfunction [10–12]. However, the mechanism by which ECH promotes the survival of cells under oxidative damage remains unclear. Here, we discuss the therapeutic effect and mechanism of action of ECH in oxidative stress-induced male infertility. We first established an oxidative stress-induced model of spermatogenesis disorder in rats and TM3 cells. Sperm number and antioxidant capacity, expression of the MAPK signaling pathway proteins, oxidative injury, and cell viability were observed.

In this study, we evaluated the effects of ECH on sperm quality, expression of MAPK proteins, and antioxidant ability of cells to provide a theoretical basis for the clinical treatment of oxidative stress-induced male infertility using ECH.

## Materials and Methods

### Animals

**Fig. 1:**
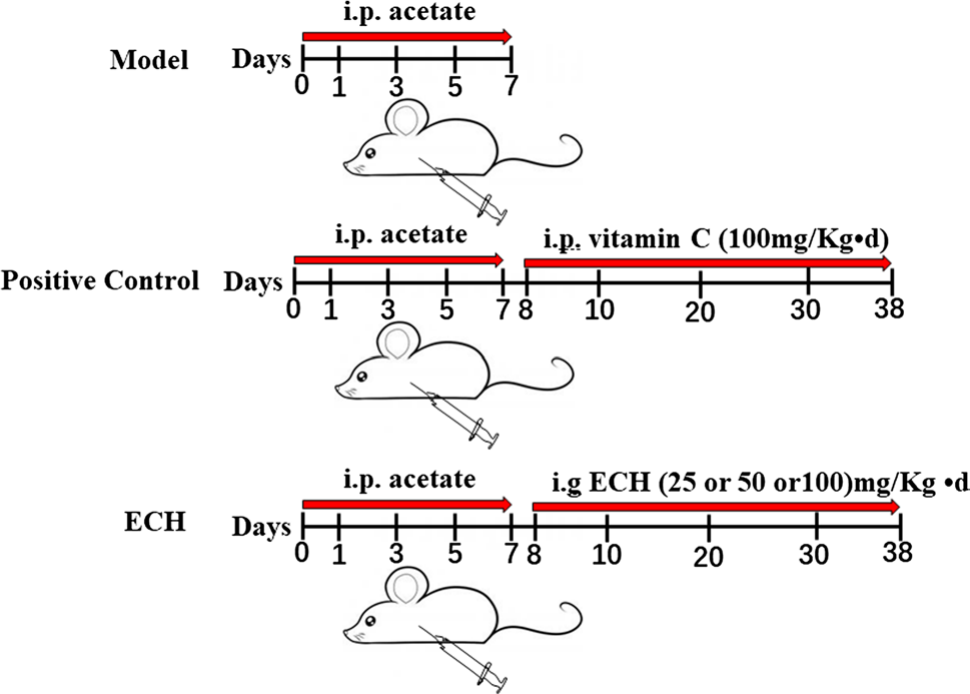
Schematic of in vivo animals assay

**Fig. 2:**
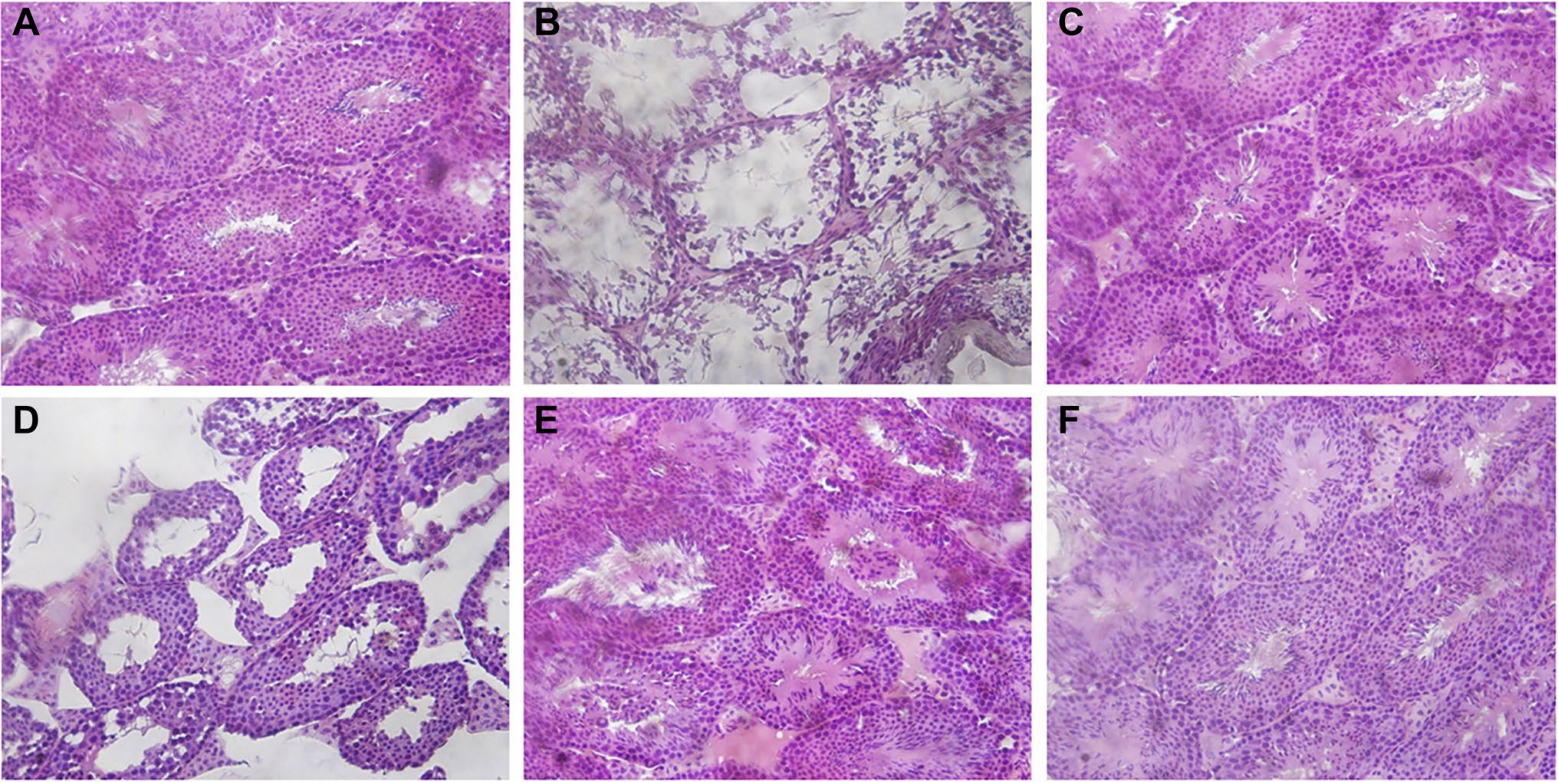
**A** in vivo experiments. **B** in vitro experiments

All animal experiments were conducted in compliance with the guidelines approved by the China Association of Laboratory Animal Care and the Institutional Animal Care Committee. Sixty adult male Wistar rats (120–180 g) were purchased from Fukang Animal Breading Center (Beijing, China) and kept at the Institutional Animal Center, Jilin Medical University (China). The rats were housed in 6 cages and acclimatized for a week. Rats were randomly divided into 6 groups: control, model, positive control (vitamin C 100 mg/kg day) [13–16], low-dose ECH (purity > 99%, 20,160,508, Sichuan victory, China (25 mg/kg day), medium-dose ECH (50 mg/kg day), and high-dose ECH (100 mg/kg day). Rats in the control group were maintained under normal feeding conditions. In addition to the control group, rats in the other groups were intraperitoneally injected with lead acetate (analytically pure, Shanghai Chemical Reagent Factory) for 7 days to generate the animal model. The positive group was intraperitoneally injected with vitamin C for 30 days. Three ECH groups were i.g. fed ECH in different dosages for 30 days, at the end of 4-week exposure and consequently after 24 h of the last administration (Fig. 1). Finally, the right testes of animals were used for hematoxylin and eosin (H&E) staining, whereas left testes were used for the index test and Western blot analysis (Fig. 2A).

### Morphological Observation of Testis

Rats (*n* = 10 in each group) were euthanized using an overdose of chloral hydrate (R00634, Beijing Leagene, China) after 4 weeks, and the right testes were enucleated and fixed in 4% paraformaldehyde (DF0134, Beijing Leagene, China) for 24 h. Fixed testicular tissues were embedded in paraffin and cut into 5-μm-thin sections of testicular tissue. Sections were stained with hematoxylin and eosin. A light microscope (Leica DFC500; Leica Microsystems, Wetzlar, Germany) was used for histopathological evaluation of tissue sections. Color micrographs were obtained at 400 × magnification.

### Sperm Quality Detection

Two rats were randomly selected from each group and their spermatozoa were obtained from the epididymis. The sperm count, sperm motility, and sperm deformity rate were evaluated using an optical microscope (DFC500; Leica Microsystems).

### Determining the Level of Glutathione, Lactate Dehydrogenase, and Superoxide Dismutase Activity in Testis

Rats (*n* = 10 in each group) were euthanized using an overdose of chloral hydrate after 4 weeks, and the right testes were enucleated (*n* = 5 in each group). Testicular samples were prepared as a 10% homogenate in 0.9% saline (Shimen, China) according to their weight using a homogenizer (Beckman, America) on ice. The homogenate was then sedimented at 1200 × *g* for 15 min, and the supernatant was collected and diluted.

Detection of glutathione (GSH) was based on its reaction with 5,5-dithio-bis-(2-nitrobenzoic acid) (DTNB) to produce a yellow compound. The level of GSH (A006-2; Nanjing Jiancheng, China) was determined at 405 nm using a colorimetric method.

Lactate dehydrogenase (LDH) catalyzes the production of pyruvic acid from lactic acid; pyruvate reacts with 2,4-dinitrophenylhydrazine to form pyruvate dinitrophenylhydrazone. The release of LDH (A020-2; Nanjing Jiancheng) could be used as a key index to detect the integrity of the cell membrane, with the corresponding value of the LDH activity being calculated at 450 nm using colorimetry.

The total activity of superoxide dismutase (SOD) (A001-1; Nanjing Jiancheng) was determined using the inhibition of the reduction of nitrotetrazolium blue (NTB) by the xanthine/xanthine oxidase system as a superoxide generator, as previously reported by Sun et al. [17]. The activity was assessed in supernatant samples incubated at 37 °C for 20 min. The corresponding value of SOD activity could be calculated at 450 nm using colorimetry.

### Cell Culture

The TM3 mouse Leydig cell line was donated by Jilin Medical University. Cells were cultured in DMEM/F-12 medium (HyClone, Thermo Fisher Scientific, Melbourne, Australia) supplemented with 5% fetal bovine serum (S-FBS-100; Scitecher, France) at 37 °C under an atmosphere of 5% CO_2_. TM3 cells were divided into 5 groups: control, model, low-dose ECH (50 μmol/L), medium-dose ECH (100 μmol/L), and high-dose ECH (200 μmol/L). Cells, with the exception of the control group, were treated with H_2_O_2_ for 4 h to generate the cell model. Then, 50 mol/L, 100 mol/L, or 200 mol/L ECH was added for 24 h (Fig. 2B).

### Cell Viability Assay

The viability of TM3 cells was detected using the MTT method. TM3 cells were seeded into 96-well plates at a density of 10^6^ cells/well. Following treatment with ECH, 20 μL of MTT (5 mg/mL) was added to each well and cells were incubated for 4 h at 37 °C. Then, 100 and 150 μL of DMSO (DH105-2; DING GUO PROSPEROUS, China) were added to each well and the 96-well plates were shaken at low speed for 10 min. Absorbance at 490 nm was measured using a microplate reader (Thermo Scientific, America).

### Determining the Lactate Dehydrogenase Activity, Levels of Glutathione Peroxidase, and Total Antioxidant Capacity of Cells

The supernatant of TM3 cells was assessed using an LDH kit (A020-2; Nanjing Jiancheng). After collecting the supernatant, tubes were centrifuged at 1000 rpm for 10 min. Absorbance at 450 nm was then measured using a microplate reader according to the manufacturer’s instructions.

The solution obtained after cell disruption was collected, and the level of glutathione peroxidase (GSH-Px) was detected using the relevant reagent kit (A005; Nanjing Jiancheng). The disrupted solution was sedimented at 1200 × *g* for 10 min, and the supernatant was collected and diluted. The corresponding level of GSH-Px was calculated using spectrophotometry (Amersham, England) at 412 nm.

The homogenate of cells was centrifuged at 12,000 × *g* for 5 min, and the supernatant was collected for measurement. The level of T-AOC in the cell homogenate was determined according to the kit instructions (A015-2–1; Nanjing Jiancheng).

### Western Blot Analysis

Total protein was extracted from tissues and cells using RIPA lysis buffer (WB-0071; DING GUO PROSPEROUS) containing proteinase and phosphatase inhibitors. Primary monoclonal antibodies included anti-p38 (1:2000; A4771; ABclonal, China), anti-phospho-p38 (1:2000; AP0526; ABclonal), anti-JNK (1:2000; A2462; ABclonal), anti-phospho-JNK (1:2000; AP0631; ABclonal), and anti-β-actin (1:5000; AC028; ABclonal). β-Actin was used as the control.

Samples containing 30 mg of protein were electrophoretically separated on 10% polyacrylamide gels containing 0.1% sodium dodecyl sulfate (SDS, DING GUO PROSPEROUS, China) and transferred to polyvinylidene difluoride (PVDF, DING GUO PROSPEROUS, China) membranes. Membranes were incubated overnight at 4 °C with one of the above primary antibodies. After membranes were washed 3 times and following the addition of goat anti-rabbit immunoglobulin G (1: 2000; AS014; ABclonal) as the secondary antibody, membranes were incubated at 25 °C for 1 h. Immunoreactive proteins were visualized using enhanced chemiluminescence (ECL; Bio-Rad, USA).

### Statistical Analysis

The ImageJ software (NIH Image, Bethesda, MD) was used to analyze Western blots. Experimental data were expressed as mean ± standard deviation (SD). A *P* value of less than 0.05 was considered statistically significant using analysis of variance (ANOVA) with the SPSS 16.0 software (SPSS) (Armonk, New York, NY, USA).

## Results

### Morphological Observation of Testis in Rats

**Fig. 3:**
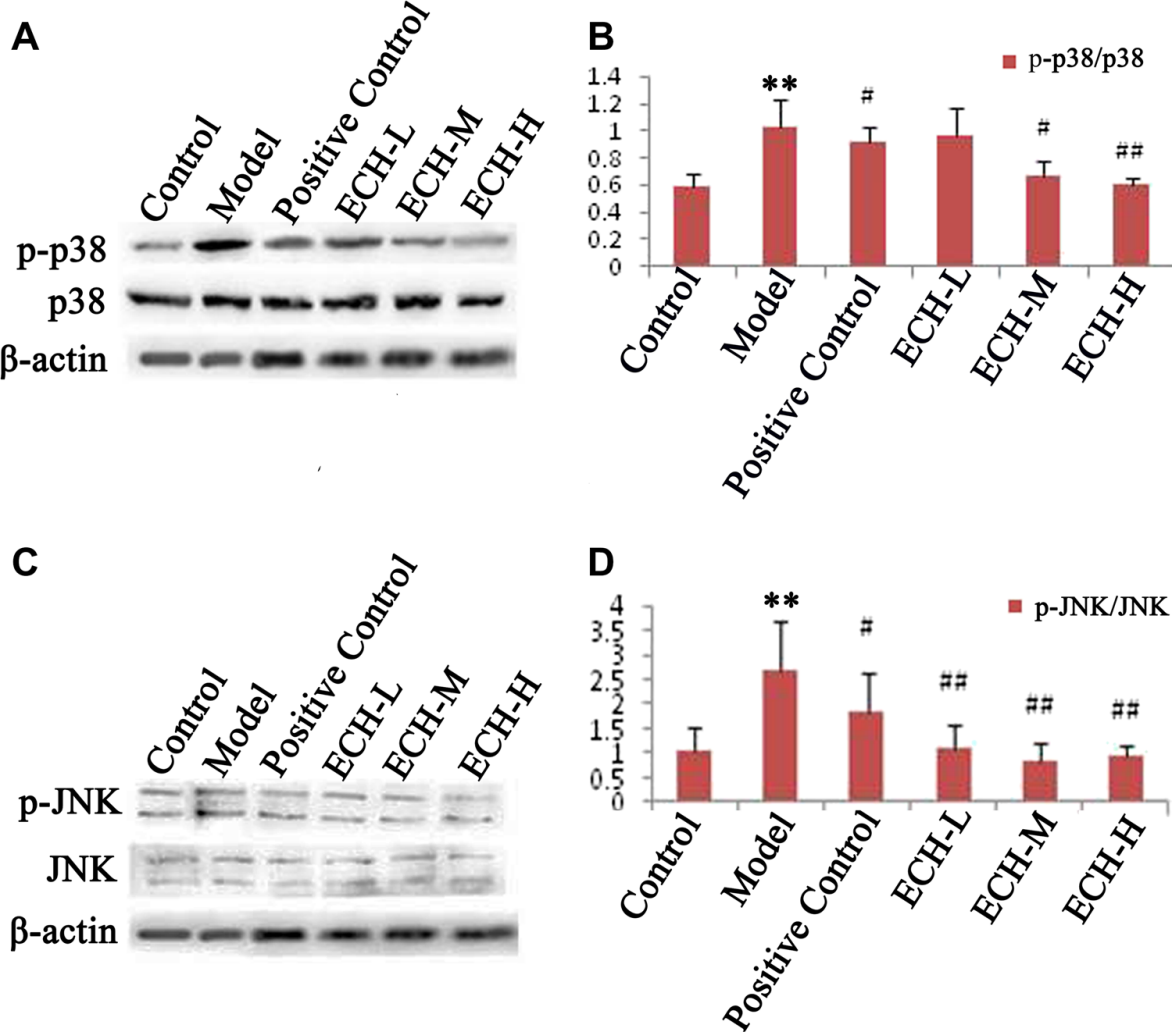
Effects of ECH on testicular histomorphology in rats with reproductive dysfunction. The upper panel shows the morphology of seminiferous tubules. Testis tissues in the control (**A**), model (**B**), positive control (**C**), ECH-L (**D**), ECH-M (**E**), and ECH-H (**F**) groups were analyzed using hematoxylin and eosin staining (magnification, × 200)

We observed changes in the testes of rats using optical microscopy and H&E staining. We found that the seminiferous tubules were atrophied; the number of layers of the seminiferous epithelium, the number of spermatogenic cells, and the number of spermatozoa in seminiferous tubules were all decreased in the testes of the model group (Fig. 3B). Following treatment with ECH, the atrophy of seminiferous tubules improved, the number of layers of the seminiferous epithelium increased, the arrangement of the epithelium was more regular, and the number of seminiferous tubules was also significantly increased. We especially noted that the seminiferous tubules were dense, the number of seminiferous epithelial cells in each layer was significantly increased, the seminiferous epithelial cells tended to be normal, and there were a large number of mature sperm in the seminiferous tubules in the high-dose and positive control groups.

### Detection of Sperm Quality

We evaluated the curative effects of ECH on the testes of mice in each group by measuring their sperm quality. As shown in Table 1, the total sperm number in the control group was significantly higher than that in the model group (*P* < 0.05). Compared with the model, the total sperm number was shown to be significantly higher in the positive control, ECH-L, ECH-M, and ECH-H groups (*P* < 0.05, *P* < 0.05, *P* < 0.05, and *P* < 0.01, respectively). We also observed that the sperm deformity rate in the model was significantly higher than that in the control group (*P* < 0.05). In contrast, the sperm deformity rate was demonstrated to be significantly decreased in the positive control, ECH-L, ECH-M, and ECH-H compared with that in the model group (*P* < 0.05). The sperm forward motility rate was found to be significantly lower in the model than in the control group (*P* < 0.05). However, the sperm forward motility rate was significantly increased in the positive control, ECH-L, ECH-M, and ECH-H compared with that in the model group (*P* < 0.01, *P* < 0.05, *P* < 0.01, and *P* < 0.01, respectively).

**Table 1:** Detection of sperm quality of rats (*n* = 10, $\overline{\mathrm{x} }$ ± s)

Groups	Total sperm number (10^6^/mL)	Sperm deformity rate (%)		Sperm forward motility rate (%)		
Control	86.27 ± 9.43	0.219 ± 0.093		67.84 ± 8.57		
Model	17.7 ± 4.1^*^	2.425 ± 0.515^*^		24.28 ± 1.16^*^		
Positive control	45.45 ± 4.85^#^	0.638 ± 0.002^#^		51.56 ± 0.77^##^		
ECH-L	28.85 ± 3.25^#^	1.108 ± 0.298^#^		29.70 ± 0.33^#^		
ECH-M	50.15 ± 17.65^#^	0.818 ± 0.228^#^		35.61 ± 0.17^##^		
ECH-H	67.45 ± 11.15^##^	0.424 ± 0.033^#^		62.6 ± 1.73^##^		
Median and confidence						
Groups	Total sperm number (10^6^/mL)		Sperm forward motility rate (%)		Sperm deformity rate (%)	
	Median	Confidence	Median	Confidence	Median	Confidence
Control	89.34	[80.43, 92.11]	0.221	[0.161, 0.277]	69.23	[62.53, 73.15]
Model	16.88	[15.16, 20.24]	2.501	[2.106,2.744]	24.43	[23.56, 25.00]
Positive control	46.96	[42.44, 48.46]	0.638	[0.637, 0.639]	51.49	[51.08, 52.04]
ECH-L	29.08	[26.84, 30.86]	0.993	[0.923, 1.293]	29.05	[29.50, 29.90]
ECH-M	49.21	[39.21, 61.09]	0.821	[0.677, 0.959]	35.43	[35.51, 35.72]
ECH-L	68.14	[60.54, 74.36]	0.419	[0.395, 0.453]	62.58	[61.53, 63.67]

Compared with control: ^∗^*P* < 0.05, ^∗∗^*P* < 0.01; compared with model: ^#^*P* < 0.05, ^##^*P* < 0.01.

### Determining the Level of Glutathione and Activity of Lactate Dehydrogenase and Superoxide Dismutase

As shown in Table 2, the levels of GSH in the control were significantly higher than those in the model group (*P* < 0.01). Compared with the model, GSH levels were significantly higher in the ECH-M and ECH-H groups (*P* < 0.01, *P* < 0.01, respectively); the GSH levels in the positive control and ECH-L groups increased but did not exhibit a significant difference. We also observed that the LDH and SOD activities in the model group were significantly lower than those in the control (*P* < 0.05). However, LDH activity in the positive control, ECH-L, ECH-M, and ECH-H groups was not significantly increased compared with those in the model group. SOD activity was shown to not be significantly increased in the positive control and ECH-M groups, whereas it was significantly increased in the ECH-H (*P* < 0.05), compared with that in the model group.

**Table 2:** Determining the level of GSH as well as activity of LDH and SOD in testis (*n* = 10, $\overline{\mathrm{x} }$ ± s)

Groups	GSH (μmol/g prot)		LDH (U/mg prot)		SOD (U/mg prot)	
Control	592.36 ± 7.71		6861.42 ± 1449.02		62.58 ± 7.71	
Model	297.36 ± 49.91^**^		4217.50 ± 1515.04^*^		48.01 ± 5.67^*^	
Positive control	342.01 ± 32.56		5551.69 ± 1606.48		49.58 ± 3.03	
ECH-L	300.17 ± 74.93		4078.81 ± 2736.41		40.90 ± 7.59	
ECH-M	598.94 ± 74.22^##^		4531.58 ± 1635.70		51.66 ± 7.71	
ECH-H	890.69 ± 191.57^##^		5473.61 ± 1575.88		62.26 ± 10.25^#^	
Median and confidence						
Groups	GSH (μmol/g prot)		LDH(U/mg prot)		SOD(U/mg prot)	
	Median	Confidence	Median	Confidence	Median	Confidence
Control	594.13	[587.58, 597.14]	6605.65	[5963.32, 7759.52]	59.73	[57.80, 67.36]
Model	287.87	[266.43, 328.29]	4235.93	[3278.49, 5156.51]	48.88	[44.50, 51.52]
Positive control	338.24	[321.83, 362.19]	5202.21	[5531.51, 5571.87]	49.44	[47.70, 51.46]
ECH-L	301.94	[253.73, 346.61]	3987.48	[4032.37, 4125.25]	39.94	[36.20, 45.60]
ECH-M	601.41	[592.94, 644.94]	4606.56	[4485.58, 4577.58]	52.19	[46.88, 56.44]
ECH-H	866.74	[771.96, 1009.42]	5640.56	[5354.88, 5592.34]	58.69	[55.91, 68.61]

Compared with control: ^∗^*P* < 0.05, ^∗∗^*P* < 0.01; compared with model: ^#^*P* < 0.05, ^##^*P* < 0.01.

### Expression of p38 and JNK in Testicular Tissues

**Fig. 4:**
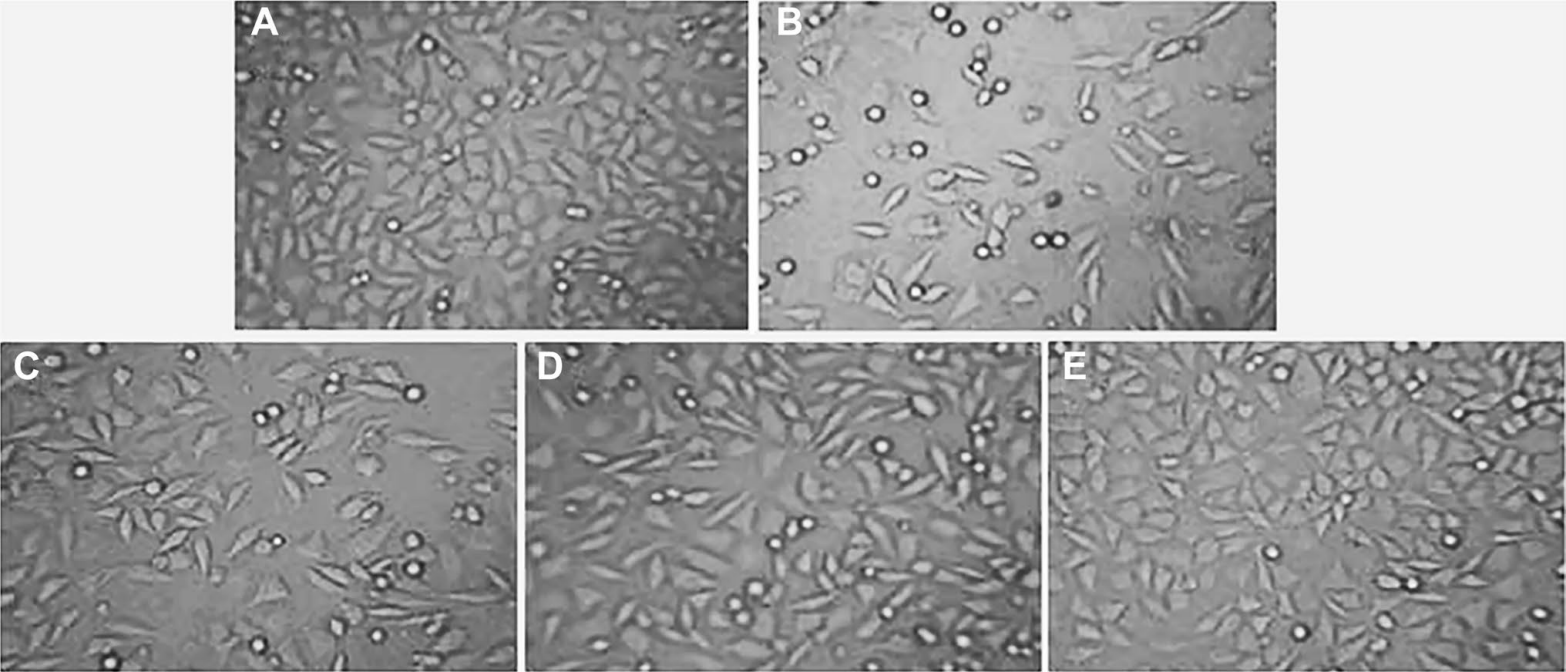
Expression of p38 and JNK in the testes of rats with spermatogenic dysfunction treated by ECH. **A**, **C** Western blotting showed that the activation of p38 and JNK in testis after spermatogenic dysfunction was statistically significant; treatment with ECH reduced these effects. **B**, **D** histogram showing the mean value ± standard error of the level of p38 and JNK proteins in testis of all groups; **compared with the control group, *P* < 0.01; #compared with the model group, *P* < 0.05; ##compared with the model group, *P* < 0.01

We analyze the expression of p38 and JNK in testicular tissues and found that the expression of p-p38 and p-JNK was significantly enhanced in the model group (Fig. 4A–B, C–D). Administration of ECH was demonstrated to partially reverse this phenomenon.

### Morphological Observation of TM3 Cells

**Fig. 5:**
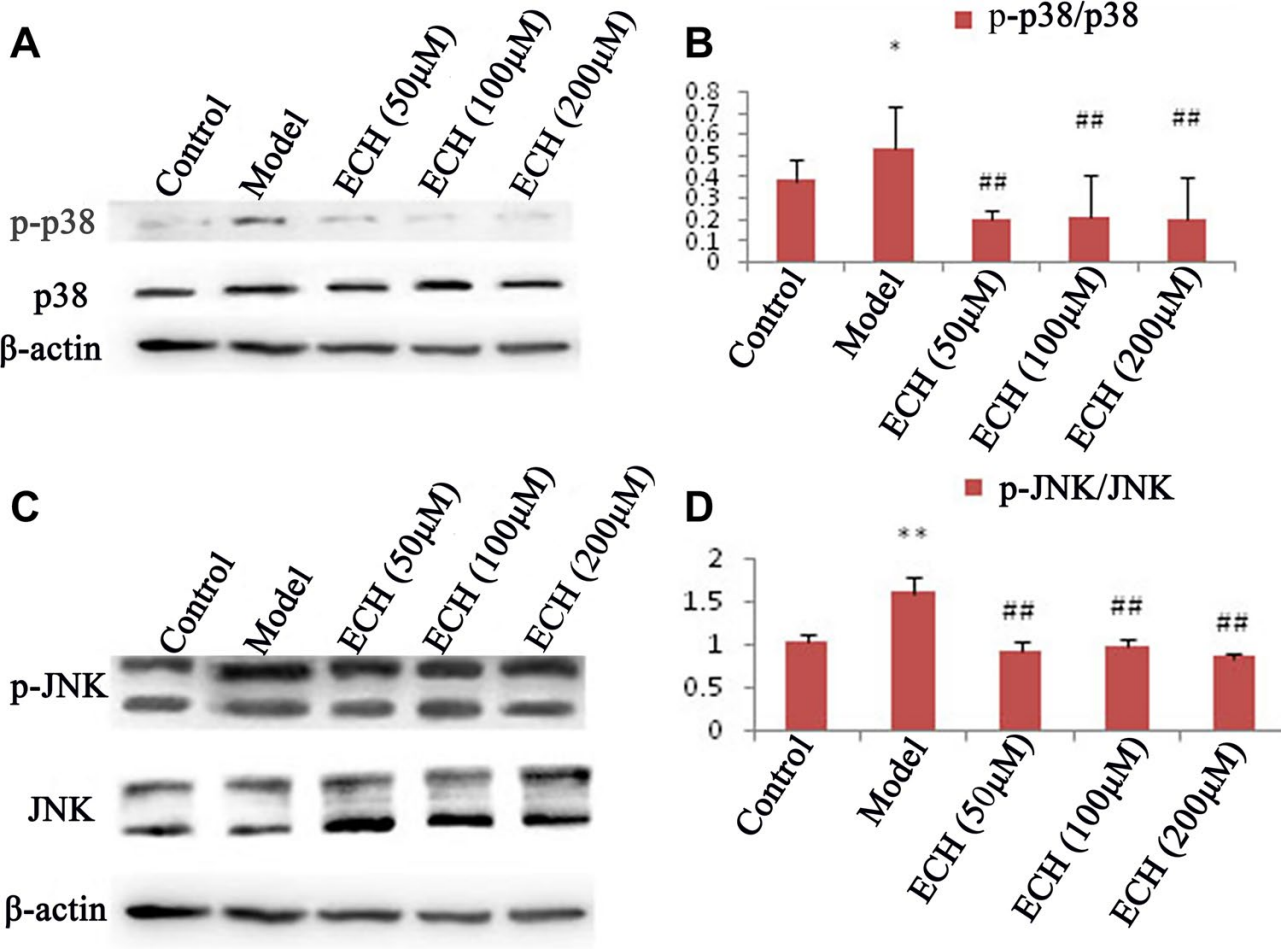
Morphological observation of TM3 cells. The upper panel shows the morphology of TM3 cells. TM3 cells of rats in the control (**A**), model (**B**), ECH (50 μmol/L) (**C**), ECH (100 μmol/L) (**D**), and ECH (200 μmol/L) (**E**) groups were microscopically observed for morphological alterations

After 24 h of cell culture, a dense and large number of TM3 cells were observed to be attached to the wall through extended triangularly shaped pseudopods, as shown in Fig. 5 (normal group). However, after injury with 400 μmol/L hydrogen peroxide, cells in the model group fell off, the cell volume was reduced, and their number was significantly decreased (*p* < 0.05). In the experimental groups, the number of cells gradually increased in an *Echinacea* dosage-dependent manner. Especially, cells in the ECH (200 μmol/L) group were shown to return to normal conditions.

### Effect of Echinacoside on Survival Rate of TM3 Cells with Oxidative Damage

We observed that the activity of TM3 cells in the model group was significantly decreased (*P* < 0.01), whereas that of cells in the 200 μmol/L ECH group was significantly increased (*P* < 0.01) compared with that in the normal group. Hence, ECH could promote the proliferation of TM3 cells (Tables 3 and 4).

**Table 3:** Effects of ECH on the activity of TM3 cells (*n* = 10, _x ± s)

**Groups**		**OD value**	**Proliferation rate (%)**
Control		0.611 ± 0.051	100 ± 8.38
Model		0.213 ± 0.027^**^	34.81 ± 4.42^**^
ECH (50 μmol/L)		0.224 ± 0.016	36.61 ± 2.61
ECH (100 μmol/L)		0.227 ± 0.018	37.13 ± 3.00
ECH (200 μmol/L)		0.279 ± 0.009^##^	45.71 ± 1.52^##^
Median and confidence			
Groups	OD value		
	Median	Confidence	
Control	0.613	[0.58, 0.64]	
Model	0.215	[0.196, 0.23]	
ECH (50 μmol/L)	0.222	[0.214, 0.234]	
ECH (100 μmol/L)	0.228	[0.117, 0.337]	
ECH (200 μmol/L)	0.279	[0.273, 0.285]	

Compared with control: ^∗^*P* < 0.05, ^∗∗^*P* < 0.01; compared with model: ^#^*P* < 0.05, ^##^*P* < 0.01.

**Table 4:** Determining the LDH activity and level of GSH-Px and T-AOC in cells (*n* = 10)

Groups	LDH(U/L)		GHS-PX (mol/L)		T-AOC (mmol/L)	
Control	66.65 ± 5.38		33.24 ± 0.052		1.139 ± 0.053	
Model	221.42 ± 0.483^**^		10.12 ± 0.014^**^		0.696 ± 0.004^*^	
ECH (50 μmol/L)	200.03 ± 1.45^##^		14.62 ± 0.083^##^		0.740 ± 0.015	
ECH (100 μmol/L)	190.44 ± 5.66^#^		20.83 ± 0.030^##^		0.871 ± 0.007^##^	
ECH (200 μmol/L)	166.36 ± 7.11^#^		20.90 ± 0.047^##^		1.013 ± 0.002^##^	
Median and confidence						
Groups	LDH(U/L)		GHS-PX (mol/L)		T-AOC (mmol/L)	
	Median	Confidence	Median	Confidence	Median	Confidence
Control	68.42	[63.32, 69.98]	33.21	[33.21,33.27]	1.141	[1.106, 1.172]
Model	220.81	[221.12, 221.72]	10.09	[10.11, 10.13]	0.695	[0.671, 0.721]
ECH-L	199.95	[199.13, 200.93]	14.68	[14.57, 14.67]	0.742	[0.731, 0.749]
ECH-M	192.83	[186.93, 193.95]	21.01	[20.81, 20.85]	0.872	[0.867, 0.875]
ECH-L	168.21	[161.95, 170.77]	21.11	[20.87, 20.93]	1.013	[0.012, 0.014]

Compared with control: ^∗^*P* < 0.05, ^∗∗^*P* < 0.01; compared with model: ^#^*P* < 0.05, ^##^*P* < 0.01.

### Western Blot Analysis

**Fig. 6:**
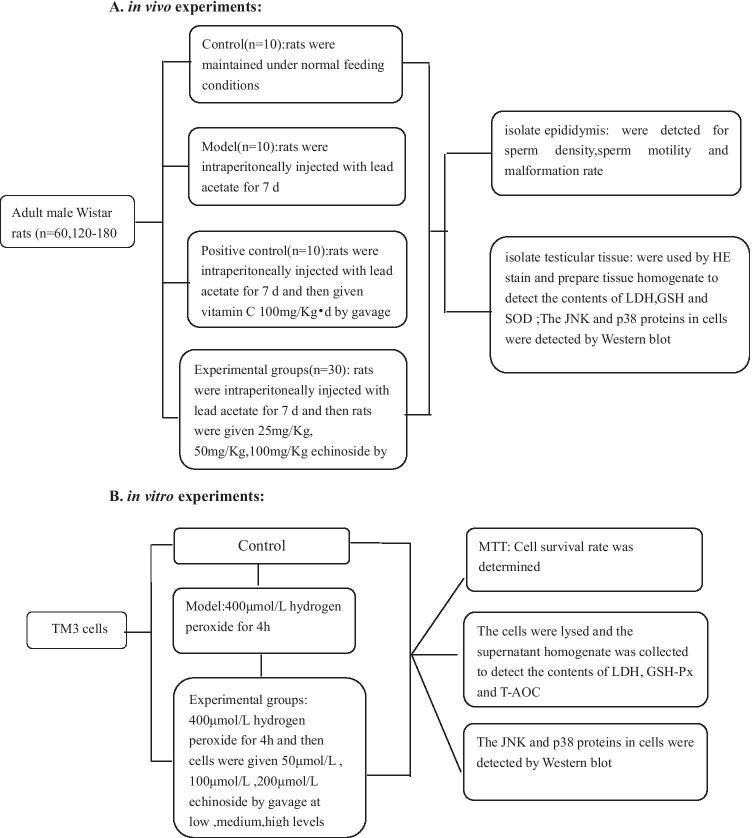
Expression of p38 and JNK in TM3 cells with spermatogenic dysfunction treated with ECH. **A**, **C** Western blotting showed that the activation of p38 and JNK in TM3 cells after spermatogenic dysfunction was statistically significant; treatment with ECH reduced these effects. **B**, **D** histogram showing the mean value ± standard error of the level of p38 and JNK proteins in TM3 cells of all groups; **compared with the control group, *P* < 0.01; ##compared with the model group, *P* < 0.01

We used Western blotting to analyze the expression of p38 and JNK in TM3 cells and found that the expression of p-p38 and p-JNK was significantly enhanced in the model group (Fig. 6A–B, C–D). However, treatment with ECH was shown to partially reverse this phenomenon.

## Discussion

Lead acetate has been used to generate models of spermatogenesis disorder in rats [18–20]. These rats were shown to be characterized by gradual weight loss, sluggish action, malaise, and sparse fur. After 7 days of intraperitoneal injection of 25 mg/kg lead acetate, the sperm activity rate and the number of sperm were decreased, the rate of sperm deformity was increased, and the testicular tissue of rats was shown to be damaged. It is reported that vitamin C(VC) has an antioxidant function, which inhibits the accumulation of ROS [17]. Vitamin C (VC) plays an efficient protective role directly or indirectly in a systemic detoxification of Pb [18]. The use of vitamin C to maintain homeostasis after lead exposure is also a relatively inexpensive and simple method. Lead poisoning may cause stimulation of lipid peroxidation, which is associated with the formation of free radicals following antioxidant depletion. Therefore, VC (100 mg/kg day) was used as the positive control group in this paper. The dose of VC in rats was determined by referring to relevant literature [19, 20].

However, after 30 days of intragastric administration of ECH, the sperm viability and sperm density increased, the deformity rate was decreased, and the testicular tissue gradually recovered. Our results showed that lead acetate significantly reduced the number of sperm, decreased the sperm activity rate, and increased the sperm deformity rate. However, after treatment with ECH, the spermatogenesis function of rat testis was gradually recovered, confirming that ECH can improve sperm quality.

Under normal conditions, reactive oxygen species (ROS) and antioxidants are in a dynamic equilibrium state in the body [21–23]. However, under pathological conditions, a large number of ROS are produced in the body, destroying the dynamic balance and leading to oxidative damage. Our study suggested that the protection of ECH against the spermatogenetic dysfunction of testes was due to the modulation of the response to oxidative stress.

Furthermore, changes in spermatogenesis-related enzyme activity might play key roles in the mechanisms of spermatogenic dysfunction. Oxidative stress and antioxidative system disorders might be important for testicular damage. Many studies have shown that the activity of testicular lactate dehydrogenase (LDH), superoxide dismutase (SOD), glutathione peroxidase (GSH-Px), and total antioxidant capacity (T-AOC) are indicators of oxidative stress [24]. Our results on the oxidative stress index showed that the levels of SOD, GSH, and LDH were decreased in the model group and were increased after the administration of ECH. This finding suggested that ECH could improve the spermatogenesis dysfunction of rats by improving the activity of antioxidant enzymes, thus inhibiting the production of lipid peroxides [25] and alleviating testicular tissue damage. Morphological observation of testes showed that the seminiferous tubules were atrophic, the number of layers of the seminiferous epithelium was significantly reduced, the spermatogenic cells decreased in number and were disorderly arranged, and the number of spermatozoa in the seminiferous tubules was decreased. However, after treatment with ECH, the atrophy of seminiferous tubules was improved, the number of layers of the seminiferous epithelium increased, the arrangement of the epithelium was more regular, and the structure of seminiferous tubules improved. These effects were especially apparent in the high-dose and positive control groups, in which the seminiferous tubules were dense, the number of seminiferous epithelial cells in each layer was significantly increased, the seminiferous epithelial cells returned to normal conditions, and the sperm in the seminiferous tubules was significantly increased.

It has been found that the MAPK signaling pathway is involved in the response to oxidative stress [26, 27]. Therefore, we used both in vivo and in vitro experiments to further explore whether the relieving effect of ECH on spermatogenesis disorder in rats was related to the MAPK signaling pathway. At present, there are three parallel MAPK signaling pathways, including the p38-mediated MAPK signaling pathway, the JNK-mediated MAPK signaling pathway, and the ERK-mediated MAPK signaling pathway. ERK mainly participates in the regulation of cell proliferation and differentiation. Various growth factor receptors and nutrition-related factor receptors require activation of ERK to complete the signal transduction process. The JNK family plays a key role in cell signal transduction induced by various stressors and is involved in oxidative stress responses. P38 is a stress-activated protein kinase similar to JNK in nature. The activator of the p38MAPK pathway is similar to that of the JNK pathway. This paper mainly discusses the changes of oxidative stress response caused by lead poisoning, so the study is mainly carried out from two pathways: JNK MAPK and p38 MAPK. Some studies have shown that p38 MAPK is one of the main members of MAPK signaling closely related to the degree of oxidative stress. The JNK family is another member of the MAPK signaling pathway and can be activated by many factors, playing an important role in the process of oxidative stress [28–30]. We performed Western blotting to detect the expression of p38, p-p38, JNK, and p-JNK in rat testes. Our results showed that the relative expression of the p-p38 and p-JNK proteins was significantly higher in the testes of the model group than in testes of the normal group. However, the relative expression of the p-p38 and p-JNK proteins was demonstrated to be decreased after treatment with *Echinacea*, suggesting that *Echinacea* might regulate the MAPK signaling pathway through the phosphorylation of p38 and JNK proteins to alleviate spermatogenic dysfunction in rats. These findings were further confirmed in vitro.

In our in vitro study, we used H_2_O_2_ on TM3 Leydig cells to generate a model of oxidative damage. We accordingly found that the protection of ECH against spermatogenetic dysfunction of testes was due to the modulation of the response to oxidative stress. Comparing the levels of LDH, GSH-Px, and T-AOC in different groups of cells, we found that ECH could enhance the antioxidant capacity of cells. Finally, we used Western blotting to detect the expression of p38, p-p38, JNK, and p-JNK in Tm3 cells. Our results showed that the relative expression of the p-p38 and p-JNK proteins was higher in the model than in the normal group. However, the relative expression of p-p38 and p-JNK proteins in cells after treatment with ECH was demonstrated to be lower than that in the model group. These in vitro findings were consistent with the results of our in vivo experiments.

## Conclusions

In summary, our study revealed that treatment with ECH alleviated spermatogenetic dysfunction of testes in male mice. Moreover, it was shown to have a remarkable impact on the expression of related proteins in Leydig cells through the activation of the MAPK signaling pathway, which might be a critical step of the mechanism underlying its therapeutic effect. These findings shed light on the potential treatment of spermatogenetic dysfunction of testes using ECH.

## Data Availability

The data generated and analyzed during this study are included in this published article and are available from the corresponding author.
